# Bessel Beams in Ophthalmology: A Review

**DOI:** 10.3390/mi14091672

**Published:** 2023-08-27

**Authors:** C. S. Suchand Sandeep, Ahmad Khairyanto, Tin Aung, Murukeshan Vadakke Matham

**Affiliations:** 1 Centre for Optical and Laser Engineering, School of Mechanical and Aerospace Engineering, Nanyang Technological University, Singapore 639798, Singapore; 2 Singapore Eye Research Institute, Singapore National Eye Centre, Singapore 169856, Singapore; 3 Duke-NUS Medical School, National University of Singapore, Singapore 169857, Singapore

**Keywords:** Bessel beam, non-diffractive beams, self-reconstruction, high-resolution imaging, ophthalmic imaging, optical sectioning, light sheet, fluorescence microscopy

## Abstract

The achievable resolution of a conventional imaging system is inevitably limited due to diffraction. Dealing with precise imaging in scattering media, such as in the case of biomedical imaging, is even more difficult owing to the weak signal-to-noise ratios. Recent developments in non-diffractive beams such as Bessel beams, Airy beams, vortex beams, and Mathieu beams have paved the way to tackle some of these challenges. This review specifically focuses on non-diffractive Bessel beams for ophthalmological applications. The theoretical foundation of the non-diffractive Bessel beam is discussed first followed by a review of various ophthalmological applications utilizing Bessel beams. The advantages and disadvantages of these techniques in comparison to those of existing state-of-the-art ophthalmological systems are discussed. The review concludes with an overview of the current developments and the future perspectives of non-diffractive beams in ophthalmology.

## 1. Introduction

High-resolution imaging of various structures in the eye is of utmost importance in ophthalmology [1,2]. For instance, high-resolution imaging of the corneal layers is essential for the detection and management of several corneal diseases [3,4]. Similarly, high-resolution imaging of the iridocorneal angle (ICA) region and the trabecular meshwork (TM) structures are important for the diagnosis of glaucoma [5,6]. High-resolution imaging of the retina is important for the detection and management of diabetic retinopathy and age-related macular degeneration [7,8]. Several ocular imaging techniques and devices have been developed over the past years for the inspection and imaging of various ocular structures [9,10,11]. Of these techniques, the most noteworthy are slit lamp biomicroscopy, scanning laser ophthalmoscopy (SLO), optical coherence tomography (OCT), in vivo confocal microscopy (IVCM), fluorescence lifetime imaging ophthalmoscopy (FLIO) and photoacoustic ophthalmoscopy (PAOM).

The slit lamp biomicroscope is one of the most common ophthalmic diagnostic tools used by clinicians for examining the eye [12,13]. Both the anterior and posterior ocular segments can be inspected using the slit lamp biomicroscope. This instrument is a combination of a lamp with an adjustable slit (as the illumination source) and a biomicroscope, fixed with the same center of rotation for parfocal movement. The slit can be adjusted in width and height and the intensity and angle can also be varied. Different sections of the eye, such as the lens and the cornea can be examined using slit lamp biomicroscopy by appropriately adjusting the slit lamp and the microscope arms. With specifically designed hand-held lenses, structures such as the ICA and the fundus can be examined as well. However, most of the commercial slit lamp biomicroscope systems lack data recording functions, as well as the resolution required for observing certain ocular structures [14].

The SLO is primarily used for the imaging of the retina and optic nerve head using a monochromatic, laser beam through a confocal raster scanning approach. SLO images are similar to those acquired via monochromatic fundus photography, but offer better quality, especially for eyes with cataracts [15]. Color/pseudo-color fundus images can be obtained by combining multiple-wavelength lasers [16,17]. This wide-field imaging device helps to identify peripheral vascular pathology in peripheral tumors, uveitis, retinal vein occlusions, and diabetic retinopathy.

OCT is a technique that has revolutionized the field of ocular imaging [18,19]. OCT enables the high-resolution visualization of both the anterior and posterior segments of the eye. OCT-based parameters have been used as standards for the management of glaucoma, and macular and retinal diseases [20,21,22]. OCT is based on low coherence interferometry where the interferometric signal is only observed from a very limited space. By performing axial and lateral scans, depth-resolved information can be generated using OCT. Initial systems developed were based on the time-domain OCT (TD-OCT) principle. Later, frequency-domain OCT (FD-OCT) systems were developed, which improved imaging speed considerably [23,24]. There are two techniques employed in FD-OCT, namely spectral-domain OCT (SD-OCT), and swept-source OCT (SS-OCT). In SD-OCT, broadband interference is acquired using a dispersive detector array, while in SS-OCT the spectral components are encoded in time using a spectral scanning light source [25]. The sensitivity and contrast of the OCT images can been improved using contrast agents [26,27]. In recent years, full-field OCT systems (FF-OCT) were developed, which unlike other OCT modalities, recorded direct en-face images, allowing rapid imaging [28,29]. Recently, OCT has been applied for OCT angiography (OCTA) and for the evaluation of age-related macular degeneration (AMD) [30].

In conventional light microscopy, light reflected from nearby structures of a point obscures the image, and the resultant background reduces the image’s contrast. Because of this, ophthalmic instruments like the slit lamp biomicroscope can only produce useful magnifications of up to about 40 times with a limited lateral resolution of about 20 μm [31,32]. Increasing the magnification further results in larger but blurrier images. IVCM is a novel tool that enables the cellular-level imaging of live ocular structures. Minsky in 1957 proposed that both imaging and illumination systems should focus to a point, creating the idea of “confocal” microscopy [33,34]. This resulted in the elimination of out-of-focus background data, leading to substantial improvements in axial and lateral resolutions. High magnifications of even up to 600 times are achievable, and the images recorded with IVCM are comparable to the images obtained via histochemical methods [32]. This made it possible for clinicians to examine cellular structures including immune, nerve, and epithelial cells in various ocular diseases.

Fluorescence lifetime imaging ophthalmoscopy is another novel technique that offers additional contrast for distinguishing different ocular fluorophores [35,36]. In general, a laser source is used for excitation, and the lifetime of the fluorescence emission is measured. This fluorescence lifetime (FLT) indicates the average amount of time a fluorophore stays in the excited state and can be used to distinguish between the statuses of different diseases. It has been shown that in the case of patients with diabetes mellitus, both with and without diabetic retinopathy, the FLTs of the eyes are prolonged in comparison to those of healthy control patients [37,38]. The FLTs in the superior-temporal areas were approximately 100 ps longer than those of healthy controls [39]. It was suggested that FLT prolongation is caused by the formation and buildup of advanced glycation end products in vascular cells, glial cells, and neurons due to diabetes [38]. These results indicate that metabolic changes in diabetic retinopathy can be detected using FLIO, even at very early stages.

PAOM is a non-invasive hybrid imaging technique capable of providing functional and anatomical characterizations of the retina with high-contrast and high-resolution [40]. It is based on the photoacoustic phenomenon, where the absorption of the optical excitation by the sample generates acoustic waves. The energy absorption undergone by the ocular tissues results in heat generation, transient thermo-elastic expansion, and ultrasonic signal production. These ultrasonic signals can be used for functional analysis and anatomical image reconstruction [40]. The time-resolved photoacoustic signals recorded can be converted into depth-resolved data (A-line data) and the raster scanning of the excitation beam can generate 3D volumetric photoacoustic images. Exogenous contrast agents can be used to improve the specificity and sensitivity of PAOM [41,42]. Several multimodal ocular imaging systems have been reported, combining PAOM with other imaging techniques such as SLO, OCT and FLIO, for the better visualization of ocular structures such as the retinal pigment epithelium, choroidal neovascularization, and choroidal and retinal vessels [43,44,45,46]. A better assessment of ocular conditions can be made possible with these multimodal platforms. Recently, deep learning techniques have been developed for PAOM to remove motion artefacts [47,48].

Though such specialized techniques exist, wide-field imaging is often the preferred imaging modality when a high temporal resolution for the field of view is required, such as in the case of in vivo live sample imaging. Most of the wide-field techniques in ophthalmology use Gaussian beams for illumination. Being a non-localized beam, Gaussian illuminations diffract, diverge and scatter during propagation through a medium. This will lead to the deterioration of the imaging signals captured, create ghost images and pose concerns about the accuracies of ocular structures and their positions being imaged [49]. Recently, several non-diffractive beams have been explored to tackle some of these issues in biomedical imaging [50,51,52,53,54,55]. These non-diffractive beams include Bessel beams, Airy beams, bottle beams, vortex beams, and lattice beams [51]. Localized, non-diffractive beams such as Bessel beams have been proven to be advantageous for imaging applications, especially when imaging structures through scattering media [56]. In addition, their potential for self-reconstruction beyond obstructions is another advantage when imaging in such situations [57,58]. These properties have resulted in improved penetration depths in dense media, reduced scattering artefacts and superior image quality in comparison with those of Gaussian beams for ocular imaging applications [2,5,59]. There have also been several techniques integrating these to achieve better capabilities. Notably, Diouf et al. recently showed speckle resistance using space–time light sheets [60]. They investigated the speckle response of a non-diffracting space–time light sheet in comparison to that of a Gaussian beam, Airy beam, and Bessel–Gauss beam, and showed that space-time light sheets exhibit better speckle resistance [60]. However, most of these non-diffractive beams, except the Bessel beam, have not yet been applied in the field of ophthalmology. This review in particular details the latest developments in Bessel beam-based ophthalmic applications and discusses future perspectives in this field.

## 2. Bessel Beams

**Figure 1:**
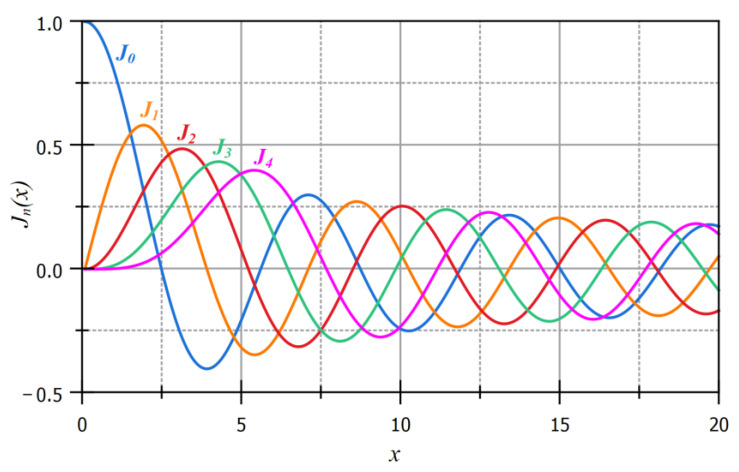
The first five orders of the Bessel function of the first kind.

Bessel beams are localized beams generated by the superposition of a set of plane waves with conically propagating wave vectors. Over a propagation distance of Δz, each of the propagating wave experiences the same phase shift, given by $k_{z}\Delta z$. The *n*th-order Bessel beam can be represented in the cylindrical coordinate system as follows [61,62]:

(1) $$E(r,\phi ,z)=A_{0}e^{\left(ik_{z}z\right)}J_{n}(k_{r}r)e^{\left(in\phi \right)}$$

where *r*, *ϕ*, and *z* are the radial, azimuthal, and longitudinal components, respectively. *A*_0_ is the electric field amplitude, *k_z_* is the longitudinal component of the wave vector, *J_n_* is the *n*th-order Bessel function of the first kind, and *k_r_* is the longitudinal component of the wave vector. The wave vector, *k*, is defined as $k=\sqrt{k_{r}^{2}+k_{z}^{2}}=2\pi /\lambda$, where *λ* is the wavelength of the Bessel beam. The zeroth-order Bessel beam has the maximum intensity at the central axis, while all the higher-order Bessel beams have the minimum intensity at the central axis. Figure 1 shows the plot of the first five orders of the Bessel function of the first kind.

Considering the Bessel beam to be a set of conically propagating plane waves, the apex angle, *θ*, is given by [63,64]

(2) $$\theta =2\times tan^{-1}(k_{r}/k_{z})$$

and the spot size of the central lobe is given by

(3) $$r_{0}=2.405/k_{r}$$

Similar to a true plane wave, a true Bessel beam would require infinite energy over infinite space, making its precise realization impossible. However, very good approximations of Bessel beams can be realized, which still possess all the desirable properties of a true Bessel beam. A very good approximation to the Bessel beam, known as the Bessel–Gauss beam can be produced experimentally via the transformation of a Gaussian beam at conical refractive surfaces [64]. The Bessel–Gauss beam is a *J*_0_ plane wave multiplied by a Gaussian beam profile [65]. The most efficient method to generate a zeroth-order Bessel–Gauss beam is through the illumination of an axicon with a Gaussian beam. The axicon can be either a refractive or diffractive type. A refractive axicon is a rotationally symmetric prism, typically with a small base angle. Upon illumination with a Gaussian beam that has a beam size smaller than the base diameter of the refractive axicon, the whole input intensity is converted into that of the Bessel beam generated. For generating higher-order Bessel beams, computer-generated diffractive axicon type holograms can be used [66,67,68].

**Figure 2:**
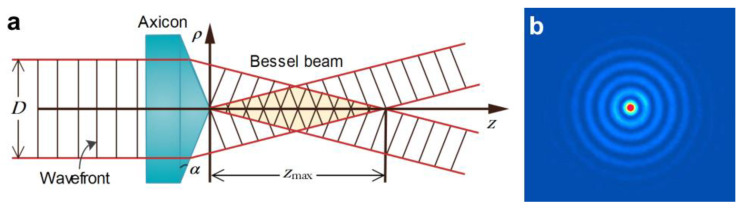
(**a**) Generation of Bessel beam using a refractive axicon. (**b**) Pseudo-colored image of the generated Bessel beam.

Figure 2a shows the schematic of zeroth-order Bessel beam generation using a refractive axicon. A pseudo-colored image of the Bessel–Gauss beam generated using a refractive axicon lens is shown in Figure 2b.

Bessel beams exhibit two outstanding features, namely the non-diffractive nature and the property of self-reconstruction. A true Bessel beam follows the equality equation given by [63]

(4) I(x,y,z≥0)=I(x,y)

This means that for a true Bessel beam, its cross-section remains unchanged during propagation (a propagation-invariant beam) [63]. However, this requires infinite energy and for approximated Bessel beams, the non-diffractive property is maintained only over a finite distance (shown as *z*_max_ in Figure 2a). The value of *z*_max_ depends on the input beam diameter (*D*), the refractive index (*n*), and the base/wedge angle (*α*) of the axicon lens, and can be approximated as follows [63]:

(5) $$z_{\max}=\frac{D}{2\alpha (n-1)}$$

Beyond *z*_max_, the Bessel beam propagates as a ring and the thickness of the ring stays invariant and is approximately equal to the radius of the input beam.

**Figure 3:**
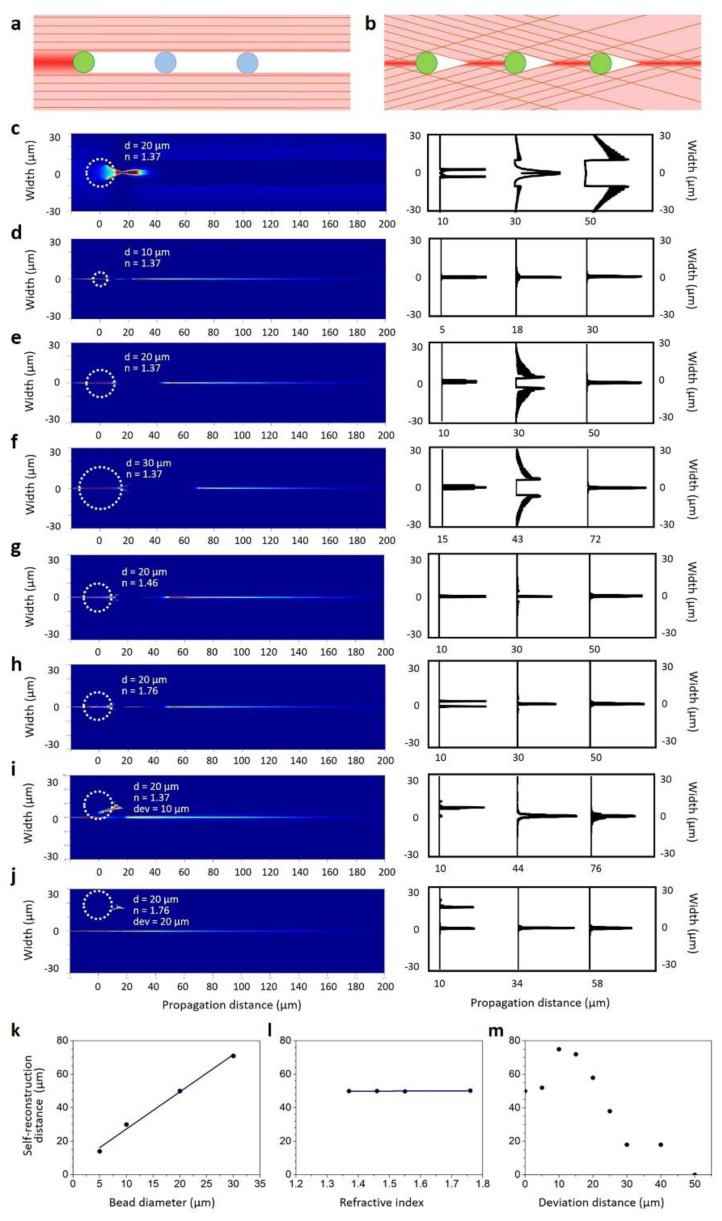
Simulations demonstrating the self-reconstruction properties of a micro-axicon (with a base diameter of 100 μm)-generated Bessel beam. Ray traces showing the (**a**) Gaussian beam and (**b**) Bessel beam encountering micro-spheres. (**c**) Gaussian beam interacting with a micro-sphere (*d* = 20 µm, *n* = 1.37, *dev* = 0 µm). (**d**–**f**) Bessel beam interacting with micro spheres of diameters of 10, 20, and 30 µm (*n* = 1.37, *dev* = 0 µm). (**g**,**h**) Bessel beam interacting with micro-spheres (*d* = 20 µm) with refractive indices of 1.46 and 1.76. (**i**,**j**) Bessel beam interacting with micro-spheres (*d* = 20 µm) displaced 10 µm and 20 µm from the axis. Self-reconstruction distances with respect to the (**k**) micro-sphere’s diameter, (**l**) micro-sphere’s refractive index, and (**m**) micro-sphere’s deviation distance from the beam axis. Reproduced from reference [64] with permission from Elsevier.

The Bessel beam’s self-reconstruction capability allows it to recover the intensity profile even after interacting with scatterers or obstructive objects. This has been demonstrated experimentally by several research groups [57,64], and is considered to be to be caused by the conical wavefronts [57,69]. The Bessel beam’s central lobe only contains a small portion of its total energy and the remaining energy is contained in the side rings. Even when the central lobe of the Bessel beam is obstructed, energy from the side lobe rings is transported to the center of the beam and the beam reforms to the initial profile after a distance, *z*_min_, beyond the obstruction. The value of *z*_min_ depends on the wavenumber and the size of the obstructing material and is given by [58,64]

(6) $$z_{\min}\approx \frac{r_{a}k}{k_{z}}$$

where *k* is the wave number, *k*_z_ is the longitudinal component of the wave vector, and *r_a_* is the radius of the obstruction measured from the beam center. The self-reconstruction property is very helpful for the high-resolution imaging of biological samples with high diffusivities that are challenging to image under Gaussian illumination. Li et al. recently reported the use of micron-sized Bessel beams generated using a 3D-printed micro-axicon lens for the high-resolution imaging of HeLa cells [64]. Figure 3 shows the detailed simulations carried out by this group on the self-healing capabilities of the micro-axicon-generated Bessel beam based on the obstructive particle’s diameter (*d*), refractive index (*n*), and deviation in position from the center of the beam (*dev*) [64]. The simulations were carried out on a micro-axicon modeled in Solidworks^®^ with a refractive index of 1.58, base diameter of 100 µm, and base angle of 20°. A collimated Gaussian beam of 532 nm wavelength was used as the illumination source. ZEMAX OpticStudio^®^ was utilized for the simulations and 100,000,000 rays were used for ray tracing. The distance from the obstruction at which the beam profile recovers to the zeroth-order Bessel function with an *R*^2^ value of 0.95 is defined as the self-reconstruction distance. These simulations show that the size and position of the obstructing particle greatly influence the self-reconstruction distance. Nevertheless, in all the cases investigated, the Bessel beam exhibited good re-construction capability. As these micro-axicons can be printed directly on the end face of optical fibers and can be used to replace bulky illumination objective lenses, they offer much higher system flexibility and unique applications in intracorporeal and hazardous environments [64].

The unique properties of Bessel beams have been utilized in several optical applications including atom guiding, optical trapping, micromanipulation, metrology and medical imaging [58]. Several advantages over systems utilizing traditional beams have been demonstrated over the past two decades. Ophthalmology is one such field where Bessel beams have been utilized for novel applications as well as for improving capabilities of current techniques. The following sections discuss the major ophthalmologic applications of Bessel beams reported in the literature in recent years.

## 3. Phakometry with Bessel Beams

**Figure 4:**
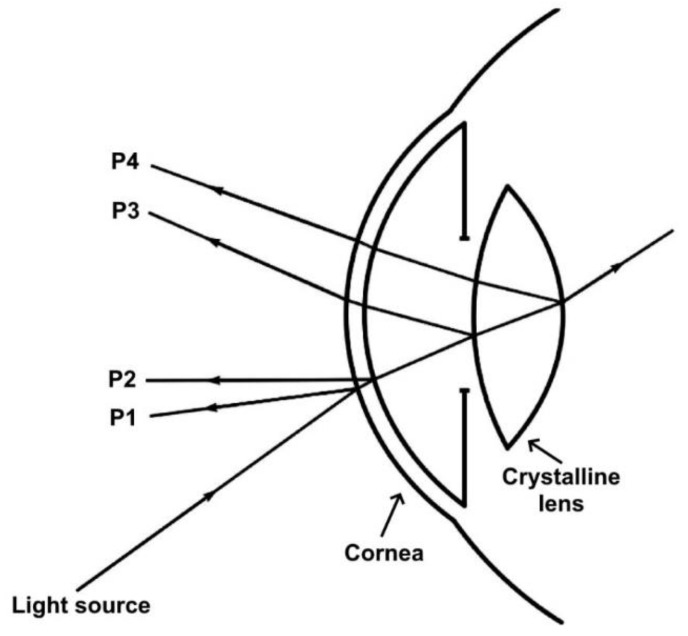
Schematic illustration of Purkinje images from ocular surfaces. Adapted from reference [70] with permission from MDPI.

The evaluation of the radii of curvature of the crystalline lens of the eye is important for several ophthalmic investigations. Phakometry is a method that typically involves photographing Purkinje images to measure the radii of curvature of crystalline lenses. Purkinje images refer to the reflections from the surfaces of the cornea and crystalline lens of the eye [70]. The first and second Purkinje images (P1 and P2) are reflections from the outer (anterior) and inner (posterior) surfaces of the cornea, respectively. The third and fourth Purkinje images (P3 and P4) are reflections caused by the outer and inner surfaces of the crystalline lens, respectively. The basis of phakometry is that the magnification of the reflected image is directly proportional to the reflecting surface’s radius of curvature. Sizes of Purkinje images P3 and P4 from the surfaces of the crystalline lens are compared to the size of Purkinje image P1 (image reflected from the cornea’s anterior surface). An illustration of Purkinje images is given in Figure 4 [70].

A major concern in phakometry is the low visibility of the Purkinje images from the anterior surface of the crystalline lens (P3) [71]. Suheimat et al. developed a Bessel beam-based phakometry system [71]. The Bessel beam was generated with the help of annular discs in front of a laser beam from a laser diode. The system was used to record Purkinje images from six participants and the results were assessed for parameters such as repeatability and Purkinje image brightness, and were compared with results obtained using a conventional phakometer. While the intraobserver repeatability for the two was similar, the interobserver repeatability was two times better with the Bessel phakometer [71]. In addition, the brightness of Purkinje images P3 and P4 was about three times higher with the Bessel phakometer. These promising results warrant further investigations in this field.

## 4. Bessel Beam-Based Light Sheet Fluorescence Microscopy (LSFM) for Ocular Imaging

The aqueous out flow system (AOS) of the eye helps in keeping the eyeball taut. In addition, it also serves the important function of transporting oxygen and nutrition to ocular tissues such as the cornea and the crystalline lens. Irregularities in the AOS can lead to an increase in intra-ocular pressure (IOP), which in turn can cause irreversible damage to the optical nerve leading to blindness [5,72]. This condition is known as glaucoma.

**Figure 5:**
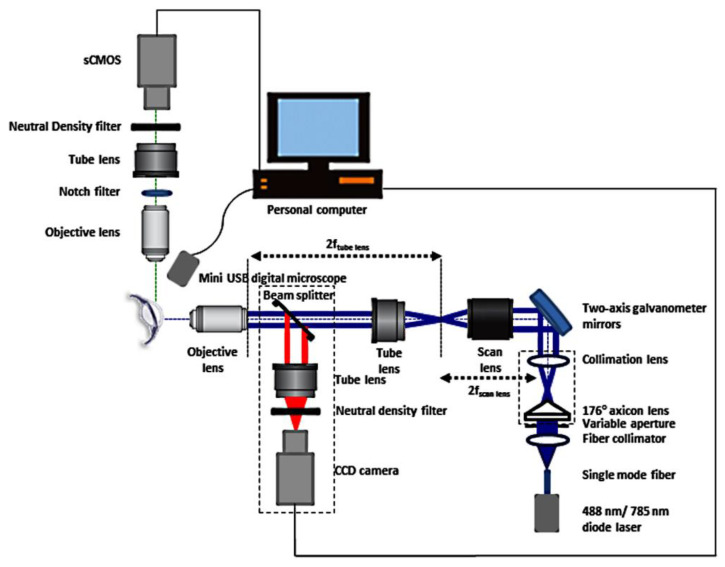
Schematic of the sequential imaging system for the high-resolution imaging of the ICA and the cornea. Reproduced from reference [72] with permission from ARVO.

Ocular structures in ICA play a major role in the regulation of the AOS. The conventional outflow of aqueous humor is through the trabecular meshwork (TM) to Schlemm’s canal located in the ICA. High-resolution imaging of the ICA would be immensely helpful to clinicians in diagnosing abnormalities in the TM, especially iris-trabecular contact (angle closure), and devising treatment strategies. The TM comprises endothelial cells, glycoproteins, elastic fibers and collagen [73]. Traditional ocular imaging techniques including gonioscopy, SLO, OCT, PAOM, ultrasound biomicroscopy (UBM), etc., could not provide the resolution and imaging contrast required to resolve individual TM structures [5,59]. In this context, Hong et. al proposed and developed a Bessel beam-based LSFM system for the high-resolution imaging of the ICA region and compared it with a Gaussian beam-based LSFM system [5,72]. The Bessel beam was produced using an axicon lens and galvano scanning mirrors were used to create the digitally scanned light sheet. Enucleated porcine eyes were used for the demonstration of the technique. The samples were stained with the fluorophore, fluorescein, by injecting 0.1 mL of 0.2% wt/vol fluorescein sodium solution into the anterior chamber of the eye. Bessel beam excitation was created using a 488 nm continuous-wave laser and the images were recorded using a scientific complementary metal oxide semiconductor (sCMOS) camera with appropriate fluorescence or notch filters. A large-working-distance (20 mm) microscope objective was used for imaging the TM. They were able to show the superiority of the Bessel beam-based system in imaging the TM, with a better signal-to-noise ratio, reduced scattering and shadowing artefacts, and higher axial resolution. The self-reconstruction property of the Bessel beam was instrumental in increasing the contrast in the scattering environment. They later reported a sequential imaging system for the high-resolution imaging of the ICA and the cornea of the eye and demonstrated in vivo imaging in New Zealand white rabbits [55,72]. Figure 5 shows the reported sequential imaging system utilizing Bessel beam-based LSFM for TM imaging [72].

**Figure 6:**
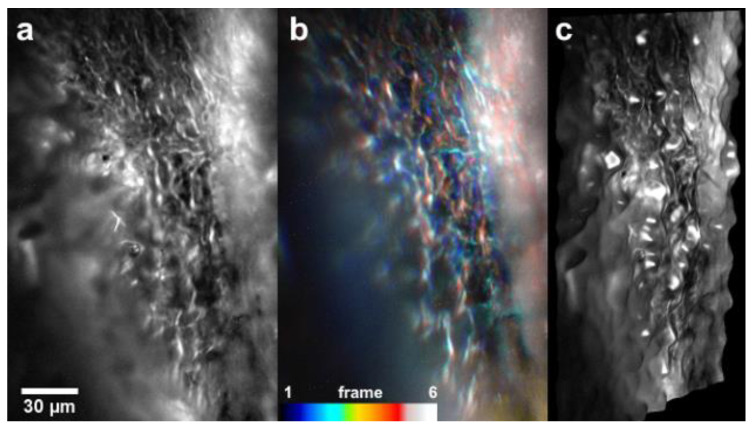
Three-dimensional visualization of the TM in an intact porcine eye. (**a**) Extended-depth-of-focus (EDF) image of the TM generated from the optical sections. (**b**) Color-coded optical sections. (**c**) Three-dimensional visualization of the TM. Reproduced from reference [59] with permission from Wiley WCH.

Suchand Sandeep et al. reported the three-dimensional (3D) visualization of the TM on an intact ex vivo porcine eye utilizing a modified version of the Bessel beam-based system [59]. The system developed had a lateral resolution of 0.78 μm and an axial resolution of 2.2 μm. The high axial resolution provided by the thin non-diffractive Bessel beam light sheet enabled the optical sectioning of the TM and the invariant focal volume provided a large field of view. Figure 6 shows the 3D visualization of the TM obtained using this Bessel beam-based LSFM system [59].

**Figure 7:**
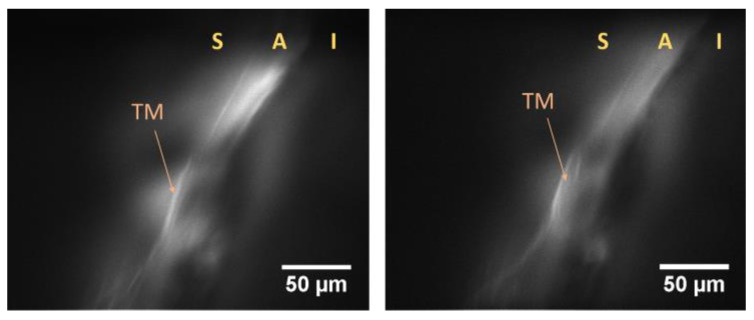
In vivo images of the TM in Wistar rats obtained using the Bessel beam-based LSFM system. S denotes the sclera, A denotes the ICA, and I denotes the iris. Reproduced from reference [74] with permission from the Optical Society.

Later, the same research group reported the in vivo imaging of the TM in live eyes of Wistar rats [74]. In order to anesthetize the Wistar rats for the measurements, 80 mg/kg of ketamine and 10 mg/kg of xylazine were administered intraperitoneally. Staining of the TM was achieved by applying 1% fluorescein eye drops. The system’s configuration was similar to the one reported in reference [59], except for the excitation objective. The use of a longer-working-distance (30 mm) apochromatic objective for exciting fluorescence from the TM provided much more flexibility during the measurements. Figure 7 displays the fluorescence images captured using the system, where the TM and the ICA regions are clearly discernible. The Bessel beam’s self-healing capability, even in a scattering environment, aids in maintaining relatively uniform illumination, resulting in improved image contrast.

Although compared to current techniques, these imaging modalities offer higher resolution and 3D visualization capabilities, their application for in vivo clinical diagnosis is still in its infancy. This is primarily because fluorescence imaging requires longer acquisition times, and in combination with the involuntary breathing motion of subjects, it can result in motion artifacts in in vivo imaging. In addition, aligning the LSFM system in a clinical setting is challenging. Nevertheless, it must be noted that only a handful of methods are available for the high-resolution imaging of the TM in vivo, and the Bessel beam-based LSFM is a very promising technique in this regard.

## 5. Retinal Imaging Using Bessel Beams

Kim et al. investigated retinal microstructures using dual-axicon-based optical frequency-domain OCT system [75]. Bessel beam illumination provided a small spot size enabling high-resolution imaging. Images were recorded from six volunteers. The lateral resolution of the en-face images recorded via ring-shaped Bessel beam illumination was compared with that under conventional Gaussian beam illumination. The lateral resolutions obtained with Bessel and Gaussian beams were 6 μm and 15 μm, respectively. The visualization of retinal and choroidal capillaries was enhanced in the Bessel beam system. In addition, spherical aberration was reduced in the case of the Bessel beam-based system. Bhattarai developed a Bessel beam-based retinal imaging system and investigated the illumination intensity reaching the retina in young (<35 years) and older (>59 years) subjects [76]. Ten volunteers were assessed from both groups. The intensities of the retinal images recorded were used to assess the illumination intensity reaching the retina. The younger group investigated did not have cataracts, while the older group contained five participants with early cataracts. In all the cases, the Bessel beam-based system provided higher retinal image intensities. The effect was more pronounced in the early cataract group. The superiority of the self-reconstruction property of the Bessel beams in scattering media was evident in the case of the cataract group [76].

Recently, Westheimer presented the modeling of retinal light distributions using truncated Bessel beams [77]. In particular, situations in which the eye’s pupil admitted only the first few lobes of the Bessel beam were investigated. The results illustrated the increased depth of focus achievable with Bessel beams and in addition, presented the generation of multi focus situations via the suitable truncation of the Bessel beam. While these investigations illustrated the higher illumination intensity achievable with Bessel beam illumination for retinal imaging, a determination of the implementation potential in actual clinical imaging systems is still lacking.

## 6. Eye Fixation Using Bessel Beams

**Figure 8:**
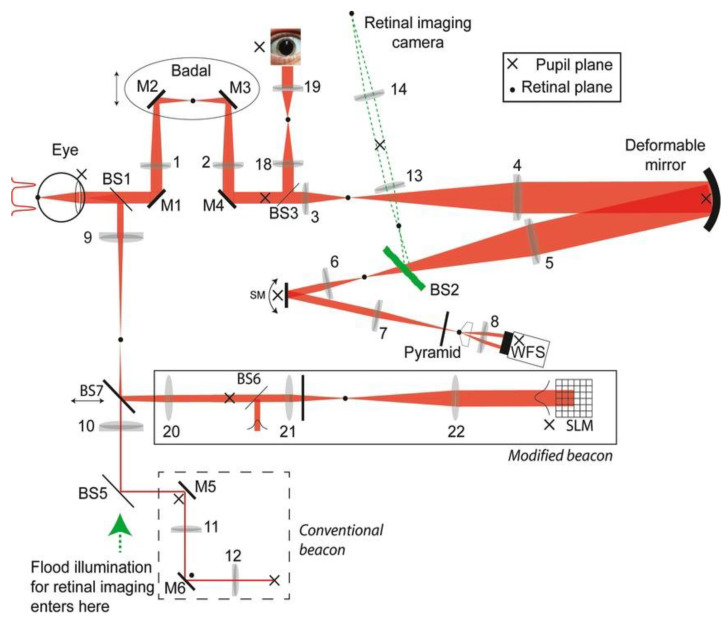
Schematic of the experimental adaptive optics (AO) system used to perform eye fixation measurements. Reproduced from reference [78] with permission from Wiley WCH.

Most of the ophthalmic imaging procedures require stable eye fixation. Involuntary eye movements such as drifts, flutters, micro-tremors and micro-saccades cause difficulties in achieving stable fixation. Several types of fixation targets such as a cross, crosshair/bull’s eye combination, circular point, etc., are used for optimizing fixation stability. Lambert et al. investigated the use of a Bessel beam as the fixation beacon in an adaptive optical system [78]. The schematic of their measurement system in shown in Figure 8. The system design wavelength was 675 nm. The probe beacons were created using a spatial light modulator (SLM). The subjects were presented with conventional (flat) and Bessel beam beacons and the RMS wavefront error and time variation of Zernike terms were evaluated. The results showed that the Bessel beam beacon receded the temporal variance considerably in comparison to that with a traditional beacon [78].

Bhattarai investigated the fixation stability of using Gaussian and Bessel laser beams and monitor-based target images [76]. The subjects were evaluated for seven fixation targets, namely a Gaussian laser beam, three-ring Bessel laser beam, four-ring Bessel laser beam, monitor-based images of the above three, and a monitor-based image of a bull’s eye/cross hair. Results from 16 participants showed that the monitor-based images of the Bessel beams provided the best fixation stability compared to laser beams, the monitor-based bull’s eye/cross hair combination and Gaussian beam [76]. The study, however, was inconclusive about the benefit of using Bessel laser beams as illumination and fixation targets. Further investigations are necessary to ascertain whether or not the Bessel beam’s propagation stability helps in improving the fixation stability.

## 7. Conclusions and Perspectives

Bessel beams offer several advantages in high-resolution imaging, target fixation and geometrical measurements for ophthalmic applications. The Bessel beam-based phakometer provided better inter-observer repeatability and brightness in comparison to those provided by Gaussian phakometers. Bessel beams were instrumental in the high-resolution LSFM imaging of TM in vivo. Optical sectioning and 3D reconstruction of the TM have also been achieved using Bessel beam-based LSFM. Retinal imaging using Bessel beams provided higher en-face resolutions and better illumination intensities in comparison to those of Gaussian beams. Bessel beams were also proven to be advantageous as fixation targets. While non-diffractive Bessel beams have been shown to possess numerous merits for high-resolution imaging, it would also be rewarding to investigate other non-diffractive beams such as Airy beams, bottle beams, lattice beams, Matheiu beams, vortex beams, etc., for ophthalmic imaging. These could be used to produce thinner light sheets to enhance axial resolutions, as well as to reduce background noise [79]. In addition, it would also be worthwhile to pursue the use of space–time light sheets, which exhibit better speckle resistance in turbid environments [60], for ophthalmic imaging applications.

OCT combined with Bessel beams has been shown to enable a large depth range of over 6 mm [80]. Several OCT modalities utilizing Bessel beams for high-resolution imaging have been reported [80,81,82,83]. However, there have not been many reports demonstrating their application in ophthalmic imaging. Bessel beam-based OCT would be a promising technique for anterior and posterior segment imaging, especially in regions with highly scattering tissues, as the self-reconstruction ability could aid in probing deeper regions.

Optical resolution photoacoustic imaging is another potential imaging technique that can have huge implications in ophthalmic imaging. Photoacoustic imaging has been shown to be very useful in imaging corneal vasculature and vessels, iris melanin, retinal vasculature, choroidal vessels and retinal pigments [40,84]. Multimodal imaging systems combining OCT and photoacoustic modalities have been demonstrated to be able to evaluate oxygen saturation in retinal vessels and the melanin-specific imaging of the retina [85,86]. The longer depth of field of Bessel beams can be utilized in photoacoustic imaging for improving the depth range, while maintaining the lateral resolution. The image degradation in the out-of-focus regions can be reduced using Bessel beam-based photoacoustic microscopy. Image processing using blind deconvolution or the Grueneisen relaxation effect has been utilized to overcome the strong side lobes of the Bessel beam [87,88,89]. Subwavelength optical resolutions with more than seven times the depth-of-field of Gaussian illumination has been reported [87]. Though optical resolution photoacoustic imaging utilizing Bessel beams has been reported in mouse ear vasculature, cerebral capillaries, red blood cells, and skin layers, it has not yet been applied for ophthalmic imaging. Further research in this field for ocular imaging could lead to potential applications such as intraoperative pathological imaging, deeper-level imaging, microcirculation monitoring, and oxygen saturation monitoring.

## Data Availability

Not applicable.
